# Pulmonary artery wall thickness and systemic sclerosis: influence of inflammation on vascular changes

**DOI:** 10.55730/1300-0144.6011

**Published:** 2025-03-24

**Authors:** Elif ALTUNEL KILINÇ, Haydar Ali CANDAN, İpek TÜRK, Çağlar ÖZMEN

**Affiliations:** 1 Division of Rheumatology, Department of Internal Medicine, Faculty of Medicine, Çukurova University, Adana, Turkiye; 2 Division of Cardiology, Faculty of Medicine, Çukurova University, Adana, Turkiye

**Keywords:** Systemic sclerosis, pulmonary artery wall thickness, Rodnan skin score, echocardiography, pulmonary hypertension, pulmonary artery pressure

## Abstract

**Background/aim:**

Systemic sclerosis (SSc) is a complex autoimmune disease marked by vascular abnormalities and fibrosis. This study aimed to compare pulmonary artery wall thickness (PAWT) among SSc patients without PAH, patients with idiopathic PAH (IPAH), and healthy controls, and to explore the clinical implications of increased PAWT in SSc patients.

**Materials and methods:**

This cross-sectional study included 60 SSc patients, 30 patients with IPAH, and 40 age- and sex-matched healthy controls. All participants underwent transthoracic echocardiographic assessment. In SSc patients, modified Rodnan skin score (mRSS), demographic characteristics, disease duration, type of skin and lung involvement, and autoantibody status were analyzed.

**Results:**

PAWT was significantly higher in the SSc group than in the control group. There was a strong correlation between PAWT and mRSS, with mRSS identified as an independent risk factor for increased PAWT. Additionally, a correlation was found between PAWT and echocardiographic parameters, particularly systolic pulmonary artery pressure (sPAP).

**Conclusion:**

PAWT is elevated in SSc patients compared to healthy controls. This may be associated with underlying vascular and fibrotic changes in SSc. Furthermore, the strong correlation between PAWT and echocardiographic markers of PAH suggests that PAWT could serve as a predictive marker for future PAH. In addition, the strong correlation between increased PAWT and mRSS suggests that elevated PAWT may represent a vascular component of SSc. Future longitudinal studies are needed to clarify the clinical significance of PAWT in SSc and to define its potential role in disease management.

## 1. Introduction

Systemic sclerosis (SSc) is a heterogeneous connective tissue disease caused by vasculopathy, fibrosis, and autoimmune inflammation, with a broad spectrum of clinical symptoms and visceral organ involvement [1]. The pathophysiology of SSc involves immune system activation, accompanied by microvascular damage, apoptosis, and a fibrotic response [2,3]. During the clinical course of the disease, progressive obliterative angiopathy occurs along with vascular remodeling. This condition primarily affects the digital arteries, pulmonary arteries, and microcirculation, resulting in various clinical manifestations [1].

The lungs are among the most commonly affected organs in SSc and typically present as pulmonary arterial hypertension (PAH) or interstitial lung disease (ILD) [4]. The prevalence of PAH among patients with SSc is estimated to be approximately 10% [5,6]. Recognizing PAH in these patients is important, as it is associated with a 50% mortality rate within 3 years of diagnosis [7]. Consequently, regular echocardiographic screening for PAH is recommended for SSc patients, regardless of symptomatology.

PAH is one of the primary causes of increased pulmonary artery wall thickness (PAWT). Elevated pressure in the pulmonary arteries stimulates endothelial cells on the intimal surface and smooth muscle cells in the medial layer, leading to proliferation and thickening of the vessel wall. This process increases mechanical stress and induces cellular responses. Besides PAH, other factors associated with increased PAWT include chronic lung diseases, cardiac disorders, obesity, sleep apnea, genetic predisposition, and inflammatory conditions [8].

This study aims to compare echocardiographically assessed PAWT among SSc patients without PAH, patients with idiopathic PAH (IPAH), and healthy controls. By focusing on SSc patients without PAH, we aim to determine whether PAWT develops independently of PAH due to inflammatory processes and whether it represents a manifestation of SSc, such as ILD or Raynaud’s phenomenon.

## 2. Materials and methods

Sixty patients with SSc who met the 2013 American College of Rheumatology/European League Against Rheumatism classification criteria were included in this cross-sectional case-control study [9]. These patients were admitted to the Çukurova University Rheumatology Clinic between January and May 2024. Additionally, 30 patients diagnosed with IPAH via right heart catheterization were recruited from the cardiology outpatient clinic, along 40 age- and sex-matched healthy controls. The study was approved by the ethics committee of Çukurova University and conducted in accordance with the Declaration of Helsinki (Date/number: 8 December 2023/139). All participants provided informed consent.

Patients over 18 years of age with a diagnosis of SSc were included in the study. Exclusion criteria included individuals under 18 years of age, those diagnosed with PAH secondary to other causes, patients with concomitant autoimmune diseases, and those with valvular heart disease affecting the pulmonary arteries. At the time of outpatient presentation, data were recorded on age, sex, disease duration, autoantibody status, presence of interstitial lung disease, modified Rodnan skin score (mRSS), pattern of skin involvement (limited/diffuse), and the Valentini disease activity index (VDAI) [10–11]. Echocardiographic examination was performed on the same day. Since no established PAWT reference value exists for SSc, a healthy control group and patients with IPAH—the primary condition associated with increased PAWT—were included for comparison. All patients in the IPAH group were diagnosed via right heart catheterization (sPAP > 25 mmHg, pulmonary vascular resistance index > 3 WU/m^2^) [12]. This group included patients over 18 years of age with no known rheumatic diseases. Echocardiograms were performed on the same day as the outpatient visit, and demographic data were documented. The healthy control group consisted of age- and sex-matched individuals without known comorbidities who attended the cardiology outpatient clinic for routine echocardiographic assessment.

**Figure 1:**
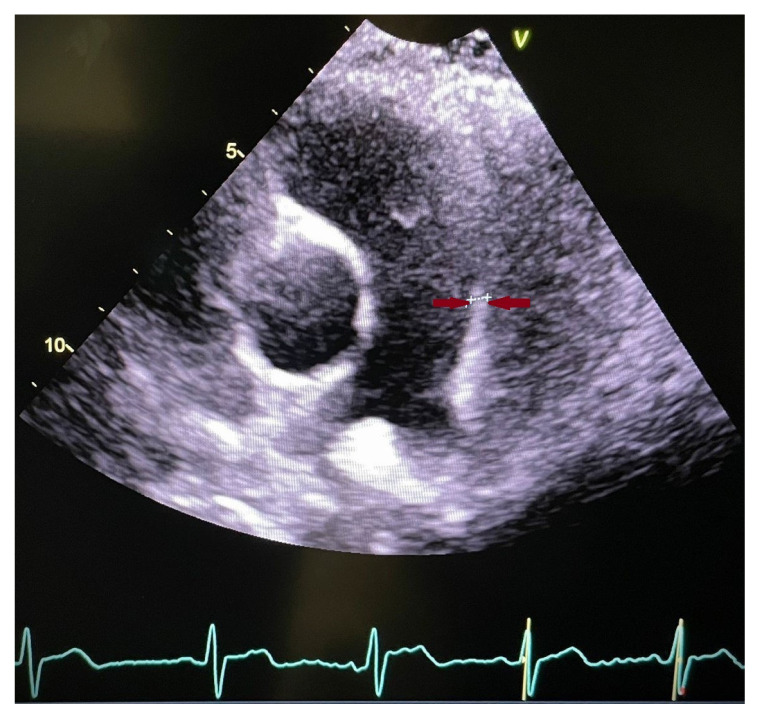
Echocardiographic measurement of pulmonary artery vessel wall thickness.

Echocardiographic examinations were performed in a blinded manner by an experienced cardiologist using a single Vivid 5 device (General Electric Healthcare, Chicago, IL, USA; serial number: 050684VS5N) for all study and control patients, in accordance with the guidelines of the American Society of Echocardiography and the European Society of Cardiovascular Imaging [13]. The assessment included right atrial (RA) size, right ventricular (RV) size, tricuspid annular plane systolic excursion (TAPSE), right ventricular systolic velocity (RVSV), right ventricular wall thickness (RVWT), systolic pulmonary artery pressure (sPAP), pulmonary artery acceleration time (PAAT), and pulmonary artery diameter (PAD), all of which are associated with PAH and reflect right ventricular function. PAWT and PAD were measured midway between the pulmonary valve and the pulmonary artery bifurcation in the parasternal short axis (Figure 1). In the apical 4-chamber view, RVWT was measured from the right ventricular free wall. sPAP was calculated by adding the estimated right atrial pressure—derived from the diameter of the inferior vena cava and its respiratory response—to the value obtained by continuous-wave Doppler across the tricuspid regurgitant jet using the modified Bernoulli equation. The upper limit of normal for sPAP was 35 mmHg; for RVWT, 5 mm; and the normal range for pulmonary artery diameter was 15–24 mm.

### 2.1. Statistical analysis

Statistical analyses were conducted using SPSS for Windows, version 25.0 (IBM Corp., Armonk, NY, USA). Descriptive statistics, including mean, standard deviation, median, minimum, maximum, frequency, and percentage values, were used to summarize the data. The Kolmogorov–Smirnov test was used to assess the distribution of the variables. To assess quantitative independent data, the independent samples t-test and the Mann–Whitney U test were used, depending on the distribution characteristics of the data. The chi-square test was performed to analyze qualitative independent data, while Fisher’s exact test was used when the assumptions for the chi-square test were not met. The Kruskal–Wallis H test was used to analyze independent quantitative data across the three groups in the study. Subsequent subgroup analyses were performed based on the results obtained from these tests. The Spearman correlation coefficient was calculated to assess correlations, represented by the symbol rho (ρ). The correlation coefficient was classified as follows: <0.25 indicates no or negligible correlation; 0.25–0.5 indicates weak to moderate correlation; 0.5–0.75 indicates strong correlation; and >0.75 indicates very strong correlation. Furthermore, receiver operating characteristic (ROC) curve analysis was used to determine effect size and to define cut-off values. Logistic regression analysis was performed to assess the diagnostic efficacy of the measurement parameters. A significance level of p < 0.05 was considered statistically significant for all analyses.

## 3. Results

The study included 60 patients with systemic sclerosis (SSc) (95% female; mean age: 56.25 ± 12 years), 30 patients with PAH (76.9% female; mean age: 50.97 ± 19 years), and 40 healthy controls (85% female; mean age: 48.6 ± 15 years). The three groups were comparable in terms of age and sex distribution (p = 0.74 and p = 0.72, respectively).

Clinical and demographic data for the SSc patients are presented in Table 1. When the SSc patients were divided into two groups—those with ILD (n = 37, 61.7%) and those without ILD (n = 23, 38.3%)—PAWT values were similar (p = 0.884). No significant difference was found in PAWT values between patients with diffuse SSc (n = 23, 38.3%) and those with limited SSc (n = 37, 61.7%) (p = 0.287). The SSc group was further classified based on autoantibody status: anti-Scl-70 antibody positive (n = 30, 50%), anticentromere antibody positive (n = 21, 35%), and seronegative or indeterminate results (n = 9, 15%). No significant differences in PAWT values were observed among these three groups (p = 0.417).

Correlation analyses between PAWT values and age, disease duration, and VDAI scores revealed no significant correlations (p = 0.216, p = 0.997, p = 0.14, respectively). However, a strong positive correlation was observed between mRSS and PAWT (r = 0.528, p < 0.001).

Further analysis of the correlation between PAWT and echocardiographic parameters within the SSc group revealed strong positive correlations with sPAP (r = 0.539, p < 0.05), RVWT (r = 0.616, p < 0.05), RA (r = 0.584, p < 0.05), RV (r = 0.438, p < 0.05), and PAD (r = 0.640, p < 0.05). Conversely, PAWT showed significant negative correlations with PAAT (r = −0.549, p < 0.05) and ejection fraction (EF) (r = −0.620, p < 0.05). No significant correlation was identified with TAPSE or RVSV (p = 0.939 and p = 0.430, respectively).

Echocardiographic evaluations including RA, RV, PAD, EF, TAPSE, RVSV, RVWT, sPAP, and PAAT were performed for SSc patients, PAH patients, and healthy controls, and the results are shown in Table 2.

**Figure 2:**
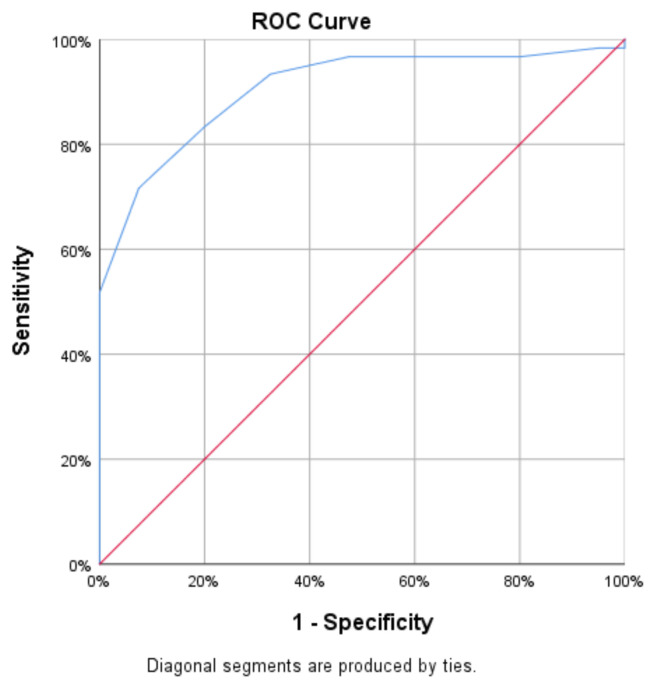
ROC analysis curve of pulmonary artery wall thickness.

ROC analysis demonstrated that PAWT effectively distinguished between healthy controls and patients with SSc (p < 0.05). The cut-off value for PAWT was established at 3.25, achieving a sensitivity of 72% (AUC [95% CI] = 0.906 [0.847–0.964]). Consequently, the SSc group was further categorized into two subsets: those with PAWT < 3.25 (n = 15, 25%) and those with PAWT > 3.25 (n = 45, 75%) (Table 3; Figure 2). Logistic regression analysis classified patients with SSc based on PAWT > 3.25 and PAWT < 3.25. Age, sex, autoantibody positivity, presence of ILD, limited versus diffuse SSc distribution, and disease duration were not identified as significant predictors of increased PAWT. However, mRSS emerged as an independent risk factor for elevated PAWT (p = 0.291, 0.362, 0.873, 0.783, 0.087, 0.483, 0.009, respectively).

## 4. Discussion

In our study, PAWT assessed via echocardiography was lower in SSc patients with PAH than in those with IPAH, but higher than in healthy controls. In SSc patients without PAH, PAWT exhibited strong positive correlations with sPAP, RVWT, and PAD, and strong negative correlations with EF and PAAT. ROC analysis indicated that PAWT effectively distinguished between SSc patients without PAH and healthy controls, demonstrating a cut-off value of 3.25.

When stratifying SSc patients without PAH based on PAWT (>3.25 vs. <3.25), variables such as presence of ILD, autoantibody subtypes (anti-Scl-70 or anti-centromere), VDAI, SSc subtype (limited vs. diffuse), and disease duration were similar across groups; however, mRSS differed significantly. Moreover, a strong positive correlation was observed between PAWT and mRSS.

In logistic regression analysis, mRSS emerged as an independent risk factor differentiating patients with PAWT > 3.25 from those with PAWT < 3.25. PAH increases PAWT through alterations in extracellular matrix deposition and vascular remodeling of distal pulmonary arteries [14]. Nonetheless, PAH is not the sole contributor to elevated PAWT; conditions such as chronic hypoxia and inflammatory diseases, including Behçet’s disease and atherosclerosis, can also lead to increased PAWT [15–17]. In PAH, increased PAWT is typically accompanied by elevations in sPAP, PAD, RV, and RVWT, and a reduction in PAAT [18].

In our study, measurements of sPAP, PAWT, PAD, RV, and RVWT were significantly higher in the IPAH group than in the SSc group. We propose that this discrepancy arises from a more pronounced hemodynamic load on the right ventricle in IPAH compared to SSc. The strong correlations observed between PAWT and sPAP, PAD, RV, and RVWT across both groups suggest that PAWT may serve as an important indicator of right ventricular loading.

Interestingly, the higher PAWT values in SSc patients without PAH, compared to healthy controls, may be attributed to the inflammatory nature of SSc, characterized by vasculopathy and fibrosis. Consequently, increased PAWT may represent one of the manifestations of the disease in patients with SSc, similar to Raynaud’s phenomenon or ILD. Furthermore, the observed correlations of PAWT with sPAP, PAD, RV, and RVWT in patients with SSc without PAH raise the question of whether PAWT could serve as a predictive marker for future PAH development. This necessitates prospective studies monitoring patients with SSc and elevated PAWT to assess the risk of PAH development.

The mRSS is a semiquantitative method used in the evaluation of skin fibrosis and is recognized as a validated outcome measure, as well as a risk factor for more severe visceral involvement in SSc. It plays a crucial role in reflecting the overall inflammatory burden of the disease [19–22]. Consistent with this, a recent study by Wyss et al. showed that ILD progression was more pronounced and overall survival was lower in patients with high mRSS scores [23].

In our study, mRSS was strongly positively correlated with PAWT and was identified as an independent risk factor for increased PAWT. These findings suggest that elevated PAWT may adversely affect pulmonary vascular structures in SSc due to inflammation and fibrosis. We propose that mRSS serves as an independent risk factor for increased PAWT and that incorporating PAWT measurements into echocardiographic evaluations of patients with high mRSS may provide valuable insights for predicting disease progression.

The presence of PHT in SSc is associated with a significantly increased mortality risk, making early detection and prediction of PHT essential. A cohort study conducted in Italy identified sPAP > 40 mmHg as the sole predictive factor for the development of PHT in patients with SSc [24]. Similarly, the Pulmonary Hypertension Assessment and Recognition of Outcomes in Scleroderma (PHAROS) cohort study found that decreased diffusion capacity of carbon monoxide, increased forced vital capacity to diffusion capacity ratio, exercise-induced hypoxia, and sPAP > 40 mmHg at initial echocardiography were all associated with future PHT development in patients with SSc [25]. The DETECT (Evidence-Based Detection of Pulmonary Arterial Hypertension in Systemic Sclerosis) study recommends assessing tricuspid jet velocity and right atrial size during echocardiographic screening of patients with SSc [26].

In light of these findings, further investigation via right heart catheterization is warranted in clinical practice when sPAP is between 35 and 40 mmHg on echocardiography. It is noteworthy that under these circumstances, PHT developed in 49% of patients [27]. Predicting the onset of PAH using parameters more sensitive than sPAP, even before any rise in sPAP, is vital for minimizing morbidity and mortality associated with SSc-related PAH. In our study, PAWT correlated with echocardiographic indicators of PAH in patients with SSc without established PAH, suggesting that PAWT assessment may be instrumental in predicting PAH development and warrants further investigation. There are insufficient studies in the literature examining the relationship between PAWT and SSc.

However, some limitations must be considered. When dividing the SSc patient population based on the cut-off value obtained from ROC analysis, 15 individuals had PAWT < 3.25 and 45had PAWT > 3.25. This imbalance may have limited the ability to optimally evaluate differences between the two groups. This study also has several strengths. While numerous studies have focused on PAH in patients with SSc, none have specifically examined PAWT or its implications for SSc. The paucity of studies on the role of PAWT in SSc pathophysiology highlights the need for in-depth investigation. Addressing this gap and demonstrating the clinical relevance of PAWT in patients with SSc constitute a key contribution of this study. As one of the first studies in this field, we systematically evaluated the effects of PAWT in patients with SSc. Our results may serve as a basis for future research and clinical applications.

In conclusion, the observation of elevated PAWT levels in patients with SSc without PAH compared to healthy controls may indicate the onset of fibrotic and vascular changes within the vessel walls. This condition may lead to vascular remodeling and potentially contribute to the development of PAH. The strong correlation between PAWT and echocardiographic indicators of PAH raises the question of whether elevated PAWT may serve as an early marker for impending PAH. To elucidate the significance of PAWT in PAH development, future studies should prospectively analyze patients with SSc and high PAWT values. Moreover, elevated PAWT in patients with SSc without PAH, along with its strong correlation with mRSS and the identification of mRSS as an independent risk factor for PAWT > 3.25, suggests that increased PAWT may reflect underlying SSc pathophysiology involving vascular damage and fibrosis. Additionally, higher PAWT may represent a clinical manifestation of the disease in patients with SSc, akin to Raynaud’s phenomenon and interstitial lung disease (ILD). Longitudinal studies revisiting these findings will enhance our understanding of the clinical significance of PAWT in SSc.

## Figures and Tables

**Table 1:** Characteristics of patients with systemic sclerosis.

**Patients, ** ***n***	60
**Age (years), mean ± SD**	56.25±12
**Female, ** ***n*** ** (%)**	57 (95)
**Duration of disease, year, median (min–max)**	8.6 (1–30)
**Modified Rodnan score mean ± SD**	18±9.6
**Valentini Disease Activity Index, median (min–max)**	0.667 (0–4.5)
**Autoantibody, ** ***n*** ** (%)**	
None*	9 (15)
**Anti-Scl-70**	30 (50)
**Anticentromere**	21 (35)
**Diagnosis, ** ***n*** ** (%)**	
**Limited systemic sclerosis**	37 (61.6)
**Diffuse systemic sclerosis**	23 (38.4)
**Interstitial lung disease, ** ***n*** ** (%)**	37 (61.6)

Anti-Scl-70: Antiscleroderma-70, SD: Standard deviation,

* Negative/unachieved.

**Table 2:** Echocardiographic findings.

	Systemic sclerosis (n=60)	Idiopathic primary arterial hypertension (n=30)	Healthy control group (n=40)	p-value
**RA**	31±3.5	47.5±10	29.8±3	<0.05^a^,^c^
**RV**	28±4	42±9.5	26±3	<0.05^a^,^c^
**EF**	61.48±5.3	61.8±4	65±2.8	<0.05^b^,^c^
**TAPSE**	22±3.5	16.6±3.5	25.5±2.6	<0.05^a^,^b^,^c^
**RVSV**	12.7±2.8	10.7±6.8	13.9±1.7	<0.05^a^,^b^,^c^
**RVWT**	3.5±0.8	5.3±1	2.8±0.2	<0.05^a^,^b^,^c^
**sPAP**	29.1 ±10.5	73±31	22.6±2.7	<0.05^a^,^b^,^c^
**PAWT**	3.35±0.47	4.08±0.4	2.8±0.2	<0.05^a^,^b^,^c^
**PAAT**	305±75	105±53	389±52.8	<0.05^a^,^b^,^c^
**PAD**	20±3.7	26.9±4	18±1.7	<0.05^a^,^c^

a for Systemic sclerosis vs Primary arterial hypertension,

b for Systemic sclerosis vs Healthy control group,

c for Primary arterial hypertension vs Healthy control group

EF: Ejection fraction, PAD: Pulmonary artery diameter, PAWT: Pulmonary artery wall thickness, PAAT: Pulmonary artery acceleration time, RA: Right atrium, RV: Right ventricle, RVSV: Right ventricular systolic velocity, RVWT: Right ventricle wall thickness, sPAP: Systolic pulmonary artery pressure, TAPSE: Tricuspid annular plane systolic excursion.

**Table 3:** Demographic characteristics of SSc patients classified according to pulmonary artery vessel wall thickness.

	PAWT >3.25 (n=45)	PAWT <3.25 (n=15)	p-value

**Interstitial lung disease, ** ***n*** ** (%)**	28(62.7)	9(60)	0.951

**Autoantibody, ** ***n*** ** (%)**			
**None**	7 (15)	2(13)	
**Anti-Scl-70**	24 (54)	6(40)	0.645
**Anti-centromere**	14 (31)	7(47)	

**Rodnan score mean ± SD**	21.5±1.9	14.45±1.3	0.008

**Valentini Disease Activity Index, median (min–max)**	0.5 (0–4.5)	0.5 (0–3.5)	0.505

**Diagnosis, ** ***n*** ** (%)**			
**Limited systemic sclerosis**	23 (51.2)	2 (13)	0.951
**Diffuse systemic sclerosis**	22 (48.8)	13 (87)	

**Duration of disease, year, median (min–max)**	8 (1–30)	10 (0–30)	0.657

PAWT: Pulmonary artery wall thickness, Anti-Scl-70: Antiscleroderma-70, SD: Standard deviation, SSc: Systemic sclerosis

## References

[1] Cutolo M Soldano S Smith V Pathophysiology of systemic sclerosis: current understanding and new insights Expert Review of Clinical Immunology 2019 15 7 753 764 10.1080/1744666X.2019.1614915 31046487

[2] Hinz B Phan SH Thannickal VJ Prunotto M Desmoulière A Recent developments in myofibroblast biology: paradigms for connective tissue remodeling The American Journal of Pathology 2012 180 4 1340 1355 10.1016/j.ajpath.2012.02.004 22387320 PMC3640252

[3] Wang Z Nakamura K Jinnin M Kudo H Goto M Establishment and gene expression analysis of disease-derived induced pluripotent stem cells of scleroderma Journal of Dermatological Science 2016 84 2 186 196 10.1016/j.jdermsci.2016.08.002 27510999

[4] Steen VD Medsger TA Changes in causes of death in systemic sclerosis, 1972–2002 Annals of the Rheumatic Diseases 2007 66 7 940 944 10.1136/ard.2006.066068 17329309 PMC1955114

[5] Mismetti V Si-Mohamed S Cottin V Interstitial lung disease associated with systemic sclerosis Seminars in Respiratory and Critical Care Medicine 2024 45 3 342 364 10.1055/s-0044-1786698 38714203

[6] Lefèvre G Dauchet L Hachulla E Montani D Sobanski V Survival and prognostic factors in systemic sclerosis-associated pulmonary hypertension: a systematic review and meta-analysis Arthritis and Rheumatism 2013 65 9 2412 2423 10.1002/art.38029 23740572

[7] Chaisson NF Hassoun PM Systemic sclerosis-associated pulmonary arterial hypertension Chest 2013 144 4 1346 1356 10.1378/chest.12-2396 24081346 PMC3787920

[8] Liu SF Nambiar Veetil N Li Q Kucherenko MM Knosalla C Pulmonary hypertension: Linking inflammation and pulmonary arterial stiffening Frontiers in Immunology 2022 13 959209 10.3389/fimmu.2022.959209 36275740 PMC9579293

[9] van den Hoogen F Khanna D Fransen J Johnson SR Baron M 2013 classification criteria for systemic sclerosis: an American college of rheumatology/European league against rheumatism collaborative initiative Annals of the Rheumatic Diseases 2013 72 11 1747 1755 10.1136/annrheumdis-2013-204424 24092682

[10] Otto CM Textbook of Clinical Echocardiography 3rd ed Philadelphia, PA, USA Elsevier Health Sciences

[11] Clements PJ Lachenbruch PA Seibold JR Zee B Steen VD Skin thickness score in systemic sclerosis: an assessment of interobserver variability in 3 independent studies The Journal of Rheumatology 1993 20 11 1892 1896 8308774

[12] Hudson M Steele R; Canadian Scleroderma Research Group (CSRG); Baron M Update on indices of disease activity in systemic sclerosis Seminars in Arthritis and Rheumatism 2007 37 2 93 98 10.1016/j.semarthrit.2007.01.005 17363039

[13] Lang RM Badano LP Mor-Avi V Afilalo J Armstrong A Recommendations for cardiac chamber quantification by echocardiography in adults: an update from the American Society of Echocardiography and the European Association of Cardiovascular Imaging Journal of the American Society of Echocardiography: official publication of the American Society of Echocardiography 2015 28 1 1 39e14 10.1093/ehjci/jev014 25559473

[14] Jandl K Radic N Zeder K Kovacs G Kwapiszewska G Pulmonary vascular fibrosis in pulmonary hypertension—The role of the extracellular matrix as a therapeutic target Pharmacology and Therapeutics 2023 247 108438 10.1016/j.pharmthera.2023.108438 37210005

[15] Humbert M Kovacs G Hoeper MM Badagliacca R Berger RMF ESC/ERS Scientific Document Group 2022 ESC/ERS Guidelines for the diagnosis and treatment of pulmonary hypertension The European Heart Journal 2022 43 38 3618 3731 10.1093/eurheartj/ehac237 36017548

[16] Fang X Chen J Hu Z Shu L Wang J Carotid baroreceptor stimulation ameliorates pulmonary arterial remodeling in rats with hypoxia-induced pulmonary hypertension Journal of the American Heart Association 2024 13 19 e035868 10.1161/JAHA.124.035868 39344593 PMC11681457

[17] Kutluğ Ağaçkıran S Sünbül M Doğan Z Kocakaya D Kayacı S Pulmonary arterial wall thickness increased in Behçet’s disease patients with major organ involvement: is it a sign of severity? Rheumatology 2023 62 3 1238 1242 10.1093/rheumatology/keac452 35944203

[18] Sandoval J Interventional therapies in pulmonary hypertension Revista Española de Cardiología (English ed) 2018 71 7 565 574 10.1016/j.rec.2018.02.002 29545075

[19] Khanna D Merkel PA Outcome measures in systemic sclerosis: an update on instruments and current research Current Rheumatology Reports 2007 9 2 151 157 10.1007/s11926-007-0010-5. 17502046

[20] Merkel PA Clements PJ Reveille JD Suarez-Almazor ME Valentini G Current status of outcome measure development for clinical trials in systemic sclerosis. Report from OMERACT 6 The Journal of Rheumatology 2003 30 7 1630 1647 12858472

[21] Clements PJ Hurwitz EL Wong WK Seibold JR Mayes M Skin thickness score as a predictor and correlate of outcome in systemic sclerosis: high-dose versus low-dose penicillamine trial Arthritis and Rheumatology 2000 43 11 2445 2454 10.1002/1529-0131(200011)43:11<2445::AID-ANR11>3.0.CO;2-Q 11083267

[22] Steen VD Medsger TA Severe organ involvement in systemic sclerosis with diffuse scleroderma Arthritis and Rheumatology 2000 43 11 2437 2444 10.1002/1529-0131(200011)43:11<2437::AID-ANR10>3.0.CO;2-U 11083266

[23] Wyss A Jordan S Graf N Carreira PE Distler J Does regression of skin thickening predict improvement of internal organ involvement and survival in patients with diffuse cutaneous systemic sclerosis? A EUSTAR analysis Arthritis Research and Therapy 2024 26 1 187 10.1186/s13075-024-03418-2 39482761 PMC11526720

[24] Iudici M Codullo V Giuggioli D Riccieri V Cuomo G Pulmonary hypertension in systemic sclerosis: prevalence, incidence and predictive factors in a large multicentric Italian cohort Clinical and Experimental Rheumatology 2013 31 2 Suppl 76 31 36 23910607

[25] Hsu VM Chung L Hummers LK Wigley F Simms R Development of pulmonary hypertension in a high-risk population with systemic sclerosis in the Pulmonary Hypertension Assessment and Recognition of Outcomes in Scleroderma (PHAROS) cohort study Seminars in Arthritis and Rheumatism 2014 44 1 55 62 10.1016/j.semarthrit.2014.03.002 24709277

[26] Hao Y Thakkar V Stevens W Morrisroe K Prior D A comparison of the predictive accuracy of three screening models for pulmonary arterial hypertension in systemic sclerosis Arthritis Research and Therapy 2015 17 1 7 10.1186/s13075-015-0517-5 25596924 PMC4332896

[27] Hsu VM Moreyra AE Wilson AC Shinnar M Shindler DM Assessment of pulmonary arterial hypertension in patients with systemic sclerosis: comparison of noninvasive tests with results of right-heart catheterization The Journal of Rheumatology 2008 35 3 458 465 18203320

